# Case report: Iliac vein rupture during endovascular stenting in radiation-induced iliac venous stenosis

**DOI:** 10.3389/fonc.2023.1166812

**Published:** 2023-05-19

**Authors:** Qilin Xiang, Jinbo Tian, Xiaoling Zhu, Chunshui He, Shan Huang

**Affiliations:** ^1^Department of Vascular Surgery, Chengdu DongLi Hospital, Chengdu, Sichuan, China; ^2^Department of Vascular Surgery, Hospital of Chengdu University of Traditional Chinese Medicine, Chengdu, Sichuan, China; ^3^Department of oncology, Sichuan Provincial People’s Hospital, Chengdu, Sichuan, China

**Keywords:** Iliac vein rupture, Iliac vein stenting, radiation-induced venous stenosis, case report, endovascular treatment

## Abstract

Only a few case reports in the medical literature describe radiation-induced iliac vein stenosis and endovascular therapy. We present a case of left external iliac vein stenosis resulting from radiotherapy for cervical cancer in which the iliac vein ruptured during the standard iliac vein stenting procedure. The emergency condition was resolved with the implantation of a covered stent and resuscitation with crystalloid and blood transfusion. The patient recovered without additional complications and was discharged eight days after endovascular therapy. At the six-month follow-up, the left lower limb edema had resolved completely, and the deep vein remained patent. This case might raise concerns regarding the potential risk of treating radiation-induced iliac venous stenosis, which may differ from that of a patient without a history of radiation therapy. Iliac vein rupture, iliac vein stenting, radiation-induced venous stenosis, case report

## Introduction

Radiation therapy is currently used to treat a range of malignancies and over fifty percent of cancer patients (1). Radiation-induced vascular disease has received an increasing amount of attention recently (2). For cancer patients following pelvic radiotherapy, severe iliac peripheral vascular disease has been described in some publications (3, 4). Compared to iliac arterial lesions, radiation-induced iliac vein stenosis is less frequently reported (4, 5). Here, we present the case of a 34-year-old woman with significant left lower extremities edema caused by iliac venous stenosis following radiation therapy for cervical cancer. After stent post-dilation, the iliac vein ruptured, necessitating the implantation of a rescue cover stent. The case revealed potential risks associated with endovascular treatment of radiation-induced iliac venous stenosis.

## Case report

**Figure 1:**
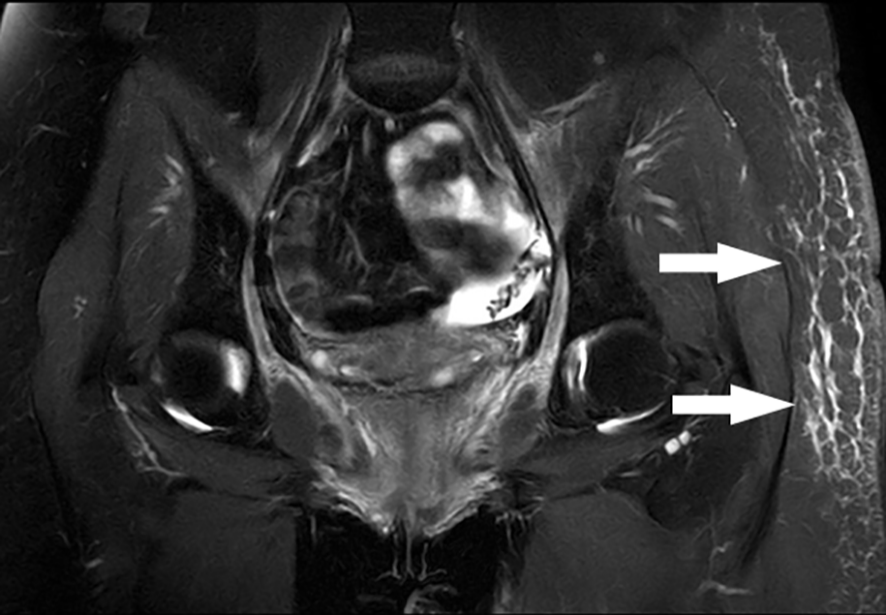
A 34-year-old female with cervical cancer developed left lower limb edema one year following surgery and pelvic radiation therapy. After administration, the pelvic MRI indicated no evidence of cancer recurrence or extrinsic compression, and left thigh subcutaneous tissue edema was observed (white arrow).

A 34-year-old female with cervical cancer was treated with surgery and postoperative whole pelvic radiation therapy at the other hospital a year ago. The cumulative radiotherapy dose is 50 Gy in 25 fractions. Three months after the radiation therapy, the patient developed progressive edema of the left lower limb, particularly in the region of the left thigh. After her admission to our clinic, the pelvic MRI revealed no indications of cancer recurrence or extrinsic compression, and left thigh subcutaneous tissue swelling was observed (**Figure 1**). Ultrasonography of the left lower extremity showed no evidence of deep venous thrombosis; however, a decrease in the velocity of the common femoral vein suggested the presence of left iliac venous stenosis.

## Procedure

**Figure 2:**
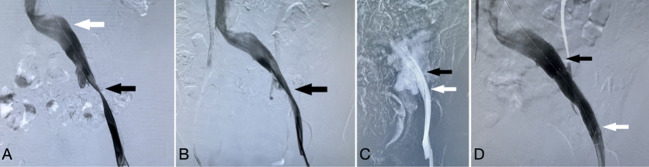
**(A)**, Venogram demonstrated significant stenosis of the left proximal external iliac vein (black arrow) and mild stenosis of the proximal common iliac vein (white arrow). **(B)** Significant recoil of stenosis of the external iliac vein after dilatation (black arrow). **(C)** Extravasation at the site of external iliac vein stenosis (black arrow) following post dilatation of the Venovo stent (white arrow). **(D)** The final venogram revealed that the covered stent (white arrow) was implanted inside the Venovo stent (black arrow) to stop the bleeding.

The patient was taken to the interventional suite under local anesthesia for additional imaging investigations and, if necessary, endovascular therapy. A 5F sheath (Terumo, Tokyo, Japan) was inserted into the left common femoral vein to perform a venogram with multiple projections observation. This revealed significant stenosis of the left proximal external iliac vein (EIV) and mild stenosis of the proximal common iliac vein (CIV) at the Iliocaval confluence (**Figure 2A**). Prior to the treatment, one bolus of 5000 units of heparin was administered intravenously. The stenoses were easily crossed utilizing a 0.035-by-260-centimeter hydrophilic-coated guidewire (Terumo, Tokyo, Japan). Afterwards, the stenoses were dilated by sequentially deploying an 8X40mm balloon (EverCross, Medtronic, Plymouth, USA) and a 12X40mm balloon (EverCross, Medtronic, Plymouth, USA). The patient had extreme pain as the EIV stenosis was dilated with the 12 mm balloon at 8 atm. During the proximal CIV stenosis dilation, there was no balloon waist and the patient did not experience discomfort. The venogram with multiple projections indicated the significant recoil of EIV stenosis after dilatation (**Figure 2B**). To provide the scaffold against recoil, a self-expanding nitinol venous stent 14X80mm (Venovo, Bard, Karlsruhe, Germany) was implanted to cover the EIV stenosis. The 12mm balloon was reintroduced to post dilate at 7 atm to prevent incomplete stent expansion. The patient again experienced excruciating pain during this step. After the balloon was deflated, the patient’s heart rate increased to 120-140 bpm, her blood pressure fell to 70-90/40-60 mmHg, and she experienced abdominal pain in the lower left quadrant. The quick angiogram revealed extravasation at the site of EIV stenosis (**Figure 2C**), and the balloon was inserted and inflated to 4 atm to stop extravasation. We immediately initiated resuscitation with crystalloid and blood transfusion to manage her hemorrhagic shock. After thirty minutes, the extravasation was still significant after deflation of the balloon. A 13.5 x 60 mm self-expanding covered stent (Fluency Plus stent graft, Bard, Karlsruhe, Germany) was implanted into the vein stent, with the proximal end just 1 cm beyond the Venovo stent (**Figure 2D**). Subsequently, the angiography confirmed the cessation of bleeding. Due to the lack of balloon contouring during the previous 12 mm balloon dilatation, the mild stenosis of the proximal CIV was not treated with stent implantation. The patient’s condition was stabilized, and the procedure was concluded. Practically immediately, the edema in the left lower extremities reduced.

**Figure 3:**
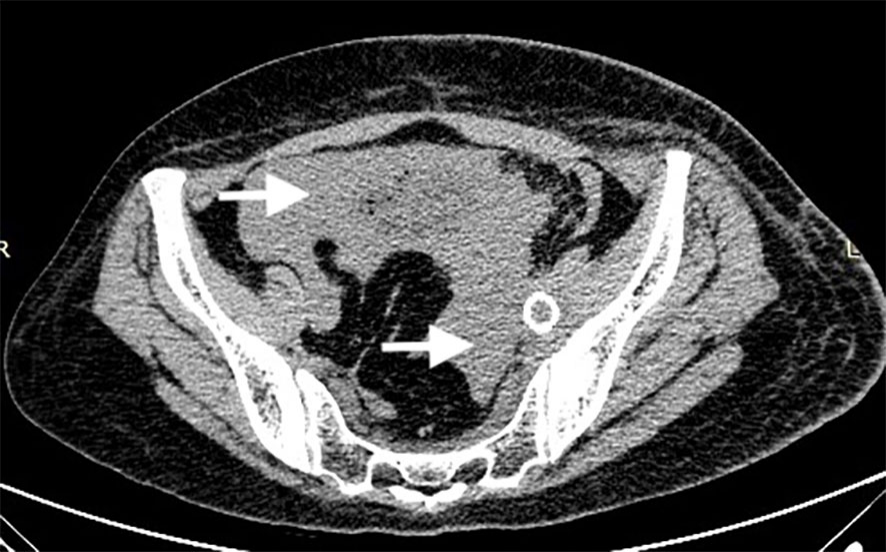
Three days after the procedure, a CT scan revealed retroperitoneal hematoma and blood and fluid accumulation in the abdominopelvic cavity. (white arrow).

Blood transfusion and anticoagulation (Nadroparin calcium 4000U, i.h., q12H) were administered postoperatively. On the third postoperative day, her hemoglobin level rose to 94 g/L, and a computed tomography (CT) scan revealed retroperitoneal hematoma and blood and fluid accumulation in the abdominopelvic cavity (**Figure 3**). On physical examination, her left lower limb had a significant reduction in tension. Eight days after the procedure, she was discharged with a prescription for rivaroxaban 20 mg orally once daily for six months and 10 mg once daily for long-term maintenance. Moreover, compression stockings were also prescribed. As postoperative follow-up, clinical and ultrasound examinations were conducted at 1, 3, 6, and 12 months, and every 12 months thereafter. At the first six-month follow-up, the left lower limb edema was completely resolved, and the left iliac, femoral, and popliteal veins were patent, as confirmed by duplex ultrasonography.

## Discussion

This case, which has not been previously reported, describes the rupture of the iliac vein during endovascular stenting in a patient with radiation-induced iliac venous stenosis and raises concerns regarding the possible risk of endovascular treatment for this type of lesion. The lower limb edema alleviation findings at 6 months showed that endovascular stenting was effective in treating patients with symptoms related to radiation-induced iliac venous stenosis.

Radiation-induced vascular disease has gained increased attention in recent years, with coronary and peripheral arterial disease receiving the most attention. The pathological alteration of human irradiation arteries revealed that fibrosis evolves over time and affects all three layers of the arterial wall, which is often indistinguishable from atherosclerotic vascular disease (2). The pathogenesis of radiation-induced venous disease is obscure, however the hypothesized disease mechanism is assumed to be similar to that of radiation-induced arteritis.

In this case, there was no evidence of local tumor recurrence, lymphadenopathy, or surrounding fibrosis, as demonstrated by a negative MRI scan during the patient’s hospitalization. This provided considerable evidence that radiation, and not tumor recurrence or external factors, was the most likely cause of EIV stenosis.

The published data on the diagnosis and management of symptomatic radiation-induced iliac venous stenosis is limited and consists primarily of case reports (4–6). In these publications, Iliac vein stenting was regarded as an important palliative option for patients who had a substantial clinical effect of venous obstruction due to its minimal invasiveness and early symptom alleviation. One publication reported arterial ischemia as a complication of venous stenting (7).

Iliac vein stenting is now regarded as a standard treatment for symptomatic lower extremity and pelvic venous outflow disorders (8, 9). In order to increase long-term stent patency while treating nonthrombotic iliac vein lesion (NIVL), several important factors must be considered during the endovascular decision-making process.

One of these concerns is the optimal stent size, which is more difficult to determine than it may appear, as Raju (10) reported using a variety of methods, including duplex scan data from healthy volunteers, patient IVUS data, the Poiseuille equation, and Young’s scaling equation. Some guidelines (11, 12) recommended slightly oversizing the stent as opposed to undersizing, with a 14mm diameter stent being the optimal size for EIV and post-dilatation procedures limited to the optimal venous outflow caliber for this diseased segment, which were deemed beneficial for long-term patency and patient safety. In this case, we followed these treatment recommendations for Iliofemoral venous obstruction. After implanting a 14 mm stent, a 12 mm balloon was used for post-dilatation; however, iliac rupture occurred, indicating that this type of lesion differs from NIVL without a history of irradiation. This raises the possibility of selecting a smaller stent for safety reasons, but patency must be closely monitored over the long term.

Increasingly, it has been proposed that intravascular ultrasound (IVUS) imaging, which permits intraluminal visualization of venous lesions, be utilized selectively in patients with suspected or confirmed iliac vein obstruction (11, 12). IVUS is an important imaging modality that allows for a more accurate assessment of vessel wall morphology and the identification of intraluminal abnormalities such as altered mural thickness, synechia, spurs, trabeculations, frozen valves, and extrinsic compression that is not always visible with conventional venous angiography (11). Along with its essential role as a diagnostic method, IVUS has become indispensable for assisting with stent sizing and appropriate stent apposition and deployment.

However, IVUS presents a number of obstacles, the most prominent of which being its unreimbursed expense in certain regions (13). This case may illustrate that it is beneficial to use IVUS in the treatment of radiation-induced venous stenosis in order to prevent a potential complication that may differ from NIVL in the absence of radiotherapy history.

This investigation was limited by the absence of conclusive evidence that iliac venous stenosis was caused by radiotherapy, despite the fact that all previous publications have relied primarily on patient histories. To establish a diagnostic protocol or consensus, more research is necessary.

## Conclusion

The utilization of iliac vein stenting has increased for the treatment of symptomatic patients with radiation-induced venous stenosis, due to its minimal invasiveness, rapid symptom relief, and low risk. This case demonstrates the potential risk of iliac vein rupture, which may be attributed to the distinct pathophysiology of radiation-induced venous stenosis compared to normal NIVL. When the lesion segment recoils severely, a smaller bare metal or IVUS usage should be considered.

## Data availability statement

The raw data supporting the conclusions of this article will be made available by the authors, without undue reservation.

## Ethics statement

The studies involving human participants were reviewed and approved by Ethics Committee of Hospital of Chengdu University of TCM. The patients/participants provided their written informed consent to participate in this study. Written informed consent was obtained from the individual(s) for the publication of any potentially identifiable images or data included in this article.

## Author contributions

QX and JT contributed equally to this work and are both considered as first author. SH and CH contributed equally to this work and are both considered as correspondence author. All authors contributed to the article and approved the submitted version.
