# Corrigendum: Case Report: Iliac vein rupture during endovascular stenting in radiation-induced iliac venous stenosis

**DOI:** 10.3389/fonc.2023.1329341

**Published:** 2023-11-07

**Authors:** Qilin Xiang, Jinbo Tian, Xiaoling Zhu, Chunshui He, Shan Huang

**Affiliations:** ^1^ Department of Vascular Surgery, Chengdu DongLi Hospital, Chengdu, Sichuan, China; ^2^ Department of Vascular Surgery, Hospital of Chengdu University of Traditional Chinese Medicine, Chengdu, Sichuan, China; ^3^ Department of oncology, Sichuan Provincial People’s Hospital, Chengdu, Sichuan, China

**Keywords:** Iliac vein rupture, Iliac vein stenting, radiation-induced venous stenosis, case report, endovascular treatment

In the published article, the names of three authors were incorrectly written as Shang Huang, Jingbo Tian and Xiaonin Zhu. The correct spelling is Shan Huang, Jinbo Tian and Xiaoling Zhu.

The authors apologize for this error and state that this does not change the scientific conclusions of the article in any way. The original article has been updated.

